# Psychiatric Polygenic Risk Scores as Predictor for Attention Deficit/Hyperactivity Disorder and Autism Spectrum Disorder in a Clinical Child and Adolescent Sample

**DOI:** 10.1007/s10519-019-09965-8

**Published:** 2019-07-25

**Authors:** Arija G. Jansen, Gwen C. Dieleman, Philip R. Jansen, Frank C. Verhulst, Danielle Posthuma, Tinca J. C. Polderman

**Affiliations:** 1 Department of Complex Trait Genetics, Center for Neurogenomics and Cognitive Research, Amsterdam Neuroscience, VU University, Amsterdam, The Netherlands; 2 Department of Child and Adolescent Psychiatry/Psychology, Erasmus University Medical Center, Rotterdam, The Netherlands; 3 Child and Adolescent Mental Health Center, Mental Health Services, Capital Region of Denmark & Faculty of Health and Medical Sciences, Department of Clinical Medicine, University of Copenhagen, Copenhagen, Denmark; 4 Department of Clinical Genetics, Section Complex Trait Genetics, Amsterdam Neuroscience, VU University Medical Center, Amsterdam, The Netherlands

**Keywords:** Polygenic risk score, Psychiatric disorders, Autism spectrum disorder (ASD), ADHD, Child behavioral checklist (CBCL), Schizophrenia

## Abstract

Neurodevelopmental disorders such as attention deficit/hyperactivity disorder (ADHD) and autism spectrum disorder (ASD) are highly heritable and influenced by many single nucleotide polymorphisms (SNPs). SNPs can be used to calculate individual polygenic risk scores (PRS) for a disorder. We aim to explore the association between the PRS for ADHD, ASD and for Schizophrenia (SCZ), and ADHD and ASD diagnoses in a clinical child and adolescent population. Based on the most recent genome wide association studies of ADHD, ASD and SCZ, PRS of each disorder were calculated for individuals of a clinical child and adolescent target sample (N = 688) and for adult controls (N = 943). We tested with logistic regression analyses for an association with (1) a single diagnosis of ADHD (N = 280), (2) a single diagnosis of ASD (N = 295), and (3) combining the two diagnoses, thus subjects with either ASD, ADHD or both (N = 688). Our results showed a significant association of the ADHD PRS with ADHD status (OR 1.6, *P* = 1.39 × 10^−07^) and with the combined ADHD/ASD status (OR 1.36, *P* = 1.211 × 10^−05^), but not with ASD status (OR 1.14, *P* = 1). No associations for the ASD and SCZ PRS were observed. In sum, the PRS of ADHD is significantly associated with the combined ADHD/ASD status. Yet, this association is primarily driven by ADHD status, suggesting disorder specific genetic effects of the ADHD PRS.

**Electronic supplementary material:**

The online version of this article (10.1007/s10519-019-09965-8) contains supplementary material, which is available to authorized users.

## Introduction

Psychiatric disorders are heritable complex traits with varying heritability estimates. At the top end of the heritability range, reported heritabilities vary from 74% for ASD (Tick et al. 2016) to 80% for ADHD (Brikell et al. 2015), and 81% for SCZ (Sullivan et al. 2003). These traits likely have a similar genetic architecture with a role for common and rare variants, including de novo mutations and copy number variants (CNV) playing an important role (Gratten et al. 2014). Common genetic variation can be captured in a polygenic signal that contains a multitude of single nucleotide polymorphisms (SNPs) from many genes (Gratten et al. 2014; Sullivan et al. 2012). Genome-wide association studies (GWAS) are a highly successful method to identify the common variants that influence these disorders (Visscher et al. 2017). GWAS reveal increasingly more significantly associated loci. These represent the most associated part of the genetic signal. The most recent GWAS for ADHD, ASD, and SCZ identified 12, 5, and 145 independent associated loci, respectively (Demontis and Walters 2017; Grove et al. 2017; Pardiñas et al. 2018).

However, given the polygenicity of disorders like ADHD and ASD, also non-significantly associated SNPs are likely to contribute to the disorder (Wray et al. 2014). Hence, it is also of interest to investigate the non-genome-wide significant component of the genetic signal.

One method to include the non-genome-wide significant component of the common genetic variation is the polygenic risk scores (PRS) approach. PRS are the sum of risk alleles weighted by their estimated effect size as determined in an independent GWAS sample, and can serve as such as an estimation of an individual’s polygenic risk (Torkamani et al. 2018; Weiner et al. 2017; Wray et al. 2014). PRS estimated from an independent sample can be used for prediction between groups (e.g., cases and controls), or for stratifying groups of people according to high or low genetic risk as defined by their PRS. For example, in a sample of children from the general population, the SCZ PRS has shown positive associations with behavioral and emotional problems in children as young as 3 years old (Jansen et al. 2017). Similarly, the ADHD PRS has been associated with attention problems in children from the general population (Groen-Blokhuis et al. 2014), and with attentional and hyperactive-impulsive traits in another general population sample (age ~ 7 year, 7 months) (Martin et al. 2014).

As previous research indicates, the common genetic burden of different psychiatric disorders partially overlaps (Mitchell 2011). To add, both ADHD and ASD, as well as SCZ, are regarded neurodevelopmental disorders (NDD) (Mullin et al. 2013; Rapoport et al. 2012) and genetic studies have shown positive genetic correlations of 0.36 for ASD/ADHD (Grove et al. 2017), 0.211 for ASD/SCZ (Grove et al. 2017), and 0.122 for ADHD/SCZ (Demontis and Walters 2017).

In addition, it was shown that the prevalence of SCZ is significantly higher in an ASD sample compared to controls (OR 3.55, 95% CI 2.08–6.05, P < 0.001), and the prevalence of ASD in an SCZ samples ranges between 3.4 and 52% compared to 1% in the general population (Zheng et al. 2018). To add, ASD and SCZ share clinical features among which social cognition (Cheung et al. 2010; *DSM 5*^2013^), while ASD and ADHD share inattention (Craig et al. 2015; *DSM 5*^2013^).

The current study adds to this literature by investigating associations of the ADHD, ASD, and SCZ PRS in a sample of children and adolescents referred to an outpatient university clinic. The children in this sample were assessed with standardized procedures generating clinical (DSM-IV) diagnoses as well as continuous rating scale scores on behavioral/emotional problems. We aim to investigate whether PRS for ADHD (Demontis and Walters 2017), ASD (Grove et al. 2017) and SCZ (Pardiñas et al. 2018) can distinguish ADHD and ASD cases from controls in this sample. Findings from genetic studies suggest a partly shared genetic diathesis underlying neurodevelopmental disorders (including SCZ, ASD and ADHD) (Bulik-Sullivan et al. 2015). We therefore hypothesized that the ADHD, ASD and SCZ PRS would be associated with the ADHD/ASD (either ASD, ADHD or both) diagnostic status. In addition, we expected both the ADHD and ASD PRS to be associated with ADHD and ASD respectively. In addition, we expected the SCZ PRS to be associated with ASD status given the genetic overlap previously reported (Autism Spectrum Disorders Working Group of The Psychiatric Genomics Consortium 2017), although conflicting results with low (Cross-Disorder Group of the Psychiatric Genomics Consortium et al. 2013) or no (Vorstman et al. 2013) genetic association between ASD and SCZ have been reported as well. As a sensitivity analysis, we aim to perform a follow up correlation analysis and subsequently a linear regression analysis with the Child Behavioral Checklist (CBCL) subscales to validate the robustness of our findings and gain additional information on the link between associated genetic signals and specific behavioral or emotional problems, given a particular clinical diagnosis.

## Methods

### Sample

#### Psychiatric outpatient sample: “Inside-Out”

A new psychiatric outpatient sample called “Inside-Out” is analyzed. Data were collected from January 2001 until January 2012 at the department of Child and Adolescent Psychiatry at the Sophia Children’s Hospital at Erasmus Medical Center in Rotterdam, resulting in a psychiatric outpatient sample. Before the first visit, parents and children received the CBCL from the Achenbach System of Empirically Based Assessment (ASEBA) (Achenbach and Rescorla 2001). In addition, DNA was extracted from saliva and genotyping was performed on the Illumina PsychChip array (see data). The procedure was approved by the ethical committee of the Erasmus Medical Center. The total Inside-Out sample comprises 1941 children diagnosed with one or more DSM-IV disorders (ASD, ADHD, Tic disorder, Obsessive Compulsive Disorder (OCD), Depression, Anxiety, Anorexia Nervosa (AN), eating disorder NOS, RETT syndrome and subcategories of mentioned disorders) and children with a delayed diagnostic status or children who did not receive a DSM diagnosis (27.9%). The diagnostic procedure consisted of an interview with parents, a semi-structured interview with the child based on the Semi-structured Clinical Interview for Children and Adolescents (McConaughy and Achenbach 2001), the Diagnostic Interview Schedule for Children IV-P (Shaffer et al. 2000) and the Autism Diagnostic Observation Schedule-Generic (Lord et al. 1989) in case of a suspected autism spectrum disorder. Diagnostic classification was done by a clinician according to the Diagnostic and Statistical Manual of Mental Disorders, fourth edition (DSM-IV). The above-mentioned procedure was part of standard clinical practice For the current study, genetic and clinical information was used of the children who received an ADHD diagnosis, no ASD co-diagnosis allowed (N = 280, age range: 3.3–18.5 years, mean: 9.06, SD: 2.66) or an ASD diagnosis, no ADHD co-diagnosis allowed (RETT excluded) (N = 295, age range: 2.5–18.3 years, mean: 9.02, SD: 3.55). In addition we used a sample of combined ADHD and ASD diagnoses where comorbidity of ADHD and ASD was allowed, adding another 113 children to this combined sample (N = 688, age range 2.5–18.5, Mean: 8.96, SD: 3.07). The target sample was diagnosed with the DSM-IV and includes many cases with Asperger and pervasive developmental disorder-not otherwise specified (PDD-NOS) diagnoses (82% of total ASD sample). ADHD and ASD co-diagnosed children (N = 113) were not included in the ADHD and ASD sample. For sample specifics see Tables 1 and 2.

**Table 1:** Sample overview

Sample	N	Reference
Discovery samples used for computation of PRS		
Discovery sample ADHD	Cases: 20.183 Controls: 35.191	Demontis et al. (2019)
Discovery sample ASD	Cases: 18.381 Controls: 27.969	Grove et al. (2019)
Discovery sample SCZ	Cases: 40.675 Controls: 64.643	Pardinas et al. (2018)

Sample	N	Additional information
Target samples used for case control studies: Inside-out (logistic regression)		
ADHD/ASD sample	688	ADHD/ASD comorbidity allowed, therefore including 113 extra children
ADHD sample	280	Subset of ADHD/ASD sample based on diagnostic status. ADHD/ASD comorbidity NOT allowed
ASD sample	295	Subset of ADHD/ASD sample based on diagnostic status. ADHD/ASD comorbidity NOT allowed
Control sample	943	NESCOG general population sample corrected for high scores on AQ and CAARS
Target sample used for sensitivity analysis: inside-out (correlations PRS-syndrome and CBCL scales)		
ADHD/ASD sample	530	Sub set of ADHD/ASD sample based on the presence of the CBCL for age 6-18 and hence, diagnostic age. ADHD/ASD comorbidity allowed

*ASD* autism spectrum disorder, *ADHD* attention deficit/hyperactivity disorder, *SCZ* schizophrenia, *CBCL* child behavioral checklist, *AQ* autism quotient, *CAARS* Conners’ Adult ADHD Rating Scale

**Table 2:** Sample description

	Sample logistic regression				Sample correlation analysis
	ADHD/ASD	ADHD	ASD	Control	ADHD/ASD^a^
N	688	280	295	943	530
Age range (mean, SD) in years	2.5–18.5 (8.96, 3.07)	3.3–18.5 (9.06, 2.66)	2.5–18.3 (9.02, 3.55)	17.0–79.0 (44.47, 13.94)	6.05–18.52 (9.7, 2.60)
Gender % male	76	75	73	38	75

ADHD/ASD = ADHD (280) + ASD (295) plus children codiagnosed with ADHD and ASD (113)

^a^Sample size differs from the sample size for the logistic regression due to CBCL 6–18 (age range) availability

#### Population-based control sample

As a control sample, we used a Dutch population sample (NESCOG, N = 943, age range: 17.0–79.0) previously described by Polderman et al. (2013). NESCOG comprises a general population and a family-based sample of which closely related individuals were excluded. Data were collected on cognitive tasks, behavioral conditions (such as ADHD and ASD symptoms), life events, personality and environmental factors, as well as genetic information. Moreover, to correct for undiagnosed ADHD status, participants scoring 3 SD above the mean on the Conners’ Adult ADHD Rating Scale (CAARS) (Conners et al. z.d.), or the Attention Problems scale of the Young Adult Self Report (YASR) (Achenbach 1997) were excluded. Participants scoring three SD above mean on the Autism Quotient (AQ) (Baron-Cohen et al. 2001) were also excluded, resulting in a final control sample of 943 participants (age range 17–79, 38% male), see Tables 1 and 2.

### Data

Genotyping of the cases and controls was performed on the same Illumina PsychChip array. The PsychChip SNP array contains HumanCore, Human Exome and custom content to accurately capture genetic variants previously linked with psychiatric disorders (https://www.illumina.com/products/by-type/microarray-kits/infinium-psycharray.html). Genetic variants in the clinical sample were filtered based on minor allele frequency (MAF < 1%), Hardy–Weinberg disequilibrium (*P* < 1 × 10^−6^) and SNP call rate (< 95%). Individuals were subsequently filtered based on relatedness (pairwise Identity-By-Descent (IBD) > 0.185), genotype and phenotypic sex mismatch, outlying heterozygosity and non-European ancestry (4 SD outside the range of the first two genetic principal components of the HapMap3 European founder population (CEU)) resulting in a clinical sample of 812 patients of which 688 are diagnosed with ADHD, ASD or both. The remaining part of the children in this sample (N = 124) are diagnosed with either Rett syndrome, Anorexia Nervosa or other eating disorders, Tourette Disorder, or other disorders. Another subset of the sample is currently being genotyped and includes children diagnosed with Anxiety Disorder, Affective Disorder or other disorders. In the control sample, SNP filtering was based on MAF (< 1%) Hardy–Weinberg disequilibrium (*P* < 0.00001) and SNP call rate (< 95%). Individual QC was based on missingness (> 5%), ancestry (within the range of 1000G CEU population on first PCs), relatedness (pairwise IBD > 0.185), gender mismatch, outlying heterozygosity and missing phenotypes.

### Sex differences in the samples

The case and control samples differed in sex distribution (cases are 75% and the controls 25% males). Therefore, we compared allele frequencies between males and females in an independent sample, GoNL (see www.nlgenome.nl for more information), by means of correlation. The Pearson correlation coefficient between the male and female allele frequencies is 0.99, removing concerns of different allele frequencies in the two samples due to sex differences.

### Polygenic risk scoring

The PRS is constructed as the sum of risk alleles weighted by their effect size. Per disorder several PRS were calculated with different *P* value inclusion thresholds (*P*-values: < 0.01, < 0.05, < 0.1, < 0.2, < 0.3, < 0.4, < 0.5, < 1). Starting from a low *P*-value threshold moving up to *P*-value 1, an optimal *P*-value threshold with the highest explained variance was identified, including the most truly associated positives. After this threshold more false positives will be included dampening the true signal. (Wray et al. 2014). Prior to our calculation of the PRS, the SNPs were pruned (LD R^2^ < 0.1, 250 kb pair window) to remove variants in LD. Polygenic scoring was performed with the software package PRSice (Euesden et al. 2015). The PRS for ASD, ADHD and SCZ were constructed using the most recent summary statistics from GWAS with the largest publicly available sample size, ADHD (Demontis and Walters 2017) (20,183 cases and 35,191 controls), ASD (Grove et al. 2017) (18,382 cases and 27,969 controls), and SCZ (Pardiñas et al. 2018) (40,675 cases and 64,643 controls). Of note, the Inside-Out and the control sample are independent samples, not included in these GWAS. After polygenic scoring the results were standardized to mean 0 and SD 1 for interpretational purposes. For the number of SNPs included in the scores see Supplementary Table S1.

### Behavioral measurements

Child emotional and behavioral problems were assessed using the Dutch version of the Child Behavior Checklist/6–18 (CBCL) (Achenbach and Rescorla 2001) filled out by the parent before the first visit to the hospital. The CBCL contains 113 problem items that can be scored on eight syndrome scales (Anxious/Depressed N_*item*_ = 13, Withdrawn/Depressed N_*item*_ = 8, Somatic Complaints N_*item*_ = 11, Social Problems N_*item*_ = 11, Thought Problems N_*item*_ = 15, Attention Problems N_*item*_ = 10, Rule Breaking Behavior N_*item*_ = 17 and Aggressive Behavior N_*item*_ = 18). Parents score each problem on a three-point scale (0: not true, 1: somewhat or sometimes true, 2: very or often true). This follow up analysis included children with a CBCL 6–18 report, completed by the parent less than a year before receiving the diagnosis. If a CBCL from within a year before diagnosis was not present the person was excluded from this part of the analysis. In all analyses, sum scores on the CBCL syndrome scales were used.

### Statistical analysis

#### Case control analysis on the association between PRS and disease status

We performed logistic regression analyses to investigate if the ADHD, ASD or SCZ PRS can distinguish between cases and controls in a sample (1) with a diagnosis of ADHD, ASD not permitted as co-diagnosis (ADHD, N = 280), (2) with a diagnosis of ASD, ADHD not permitted as co-diagnosis (ASD, N = 295), and (3) combining the first two samples, thus subjects with either ASD, ADHD or both (ADHD/ASD, N = 688). For each PRS, eight different SNP inclusion thresholds were tested. All *P*-values were corrected for multiple testing by means of Bonferroni correction (72 tests: three samples (ADHD, ASD, ADHD/ASD), three PRS (ADHD, ASD, SCZ), eight PRS thresholds (0.01, 0.05, 0.1, 0.2, 0.3, 0.4, 0.5, 1) per disorder). To account for population stratification we included eight principal components (PCs). The PCs were calculated based on the pruned data with Eigensoft (Price et al. 2006) (version 3.0) software. Additionally, sex was added as a covariate. Age was not added as a covariate as all cases are children and all controls are adults.

#### Sensitivity analysis: correlation and association between CBCL syndrome scales and PRS

We aim to provide additional evidence for the significant association of the PRS and the disorders as measured by the CBCL score severity. Given statistical power, we tested the association with symptom severity only in the combined ADHD/ASD sample by calculating the correlation between the significantly associated PRS (i.e., ADHD) and the syndrome scales of the CBCL. Age was added as a covariate in addition to the previously used eight PCs and sex. All analyses were performed in IBM SPSS statistics 21.

## Results

### Case control analysis on the association between PRS and disorder status

**Fig. 1:**
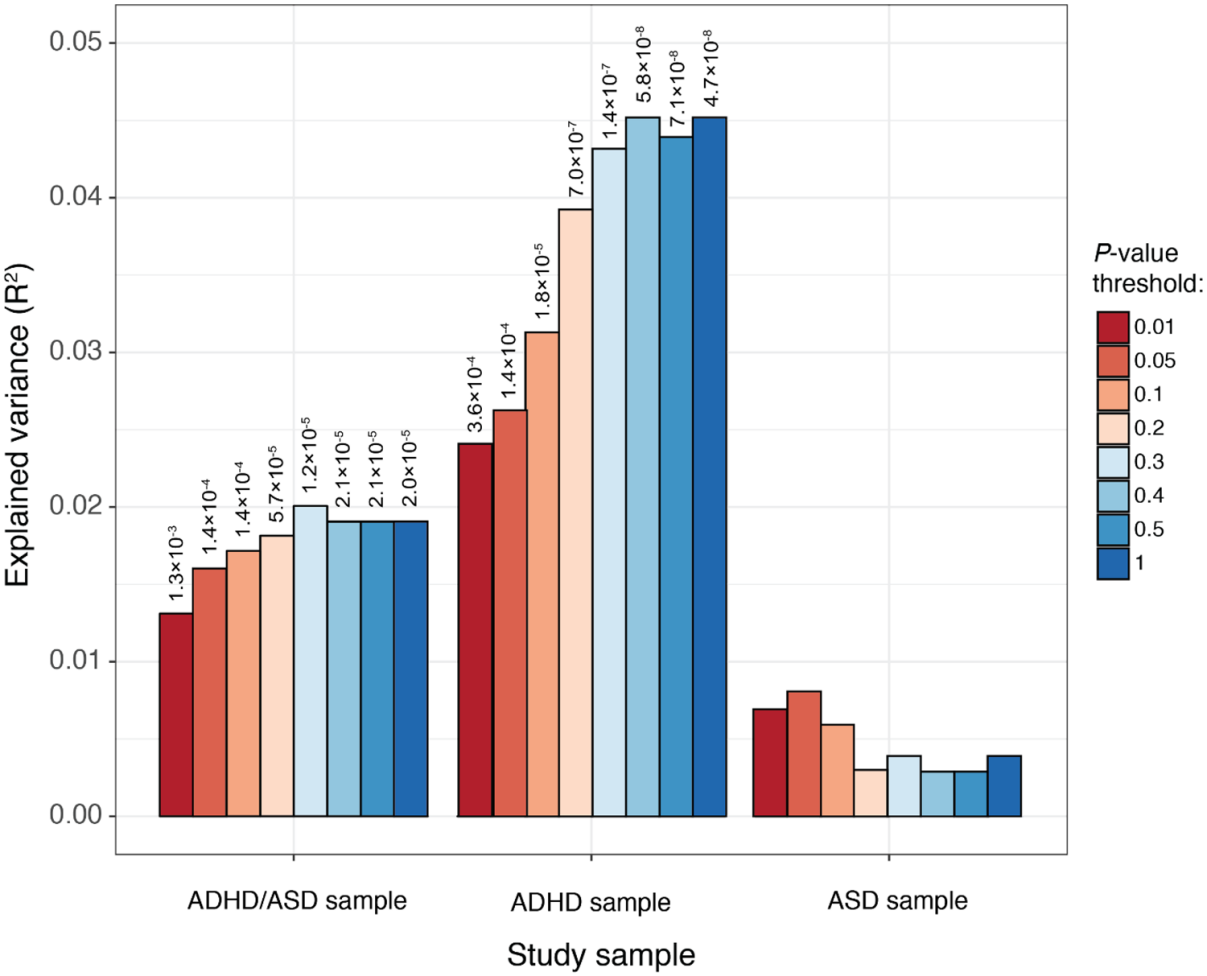
Variance explained (Nagelkerke R^2^) by the ADHD PRS. All SNP inclusion *P*-value thresholds are shown from low to high (0.01, 0.05, 0.1, 0.2, 0.3, 0.4, 0.5, 1). *P*-values are shown on top of each bar and are Bonferroni corrected

The ADHD PRS showed significant associations before multiple testing correction with disorder status in all three samples (Table 3). As shown in Fig. 1, all ADHD PRS *P*-value thresholds remained significant after Bonferroni multiple testing correction in both the combined ADHD/ASD and ADHD sample, but not the ASD sample. The most stringent *P*-value threshold of 0.01 generated a positive association in the ADHD/ASD sample OR 1.28 (*P* = 1.3 × 10^−3^), and ADHD sample OR 1.4 (*P* = 3.6 × 10^−4^). The most optimal *P*-value threshold as defined by explained variance, OR and P-value was 0.3 for the ADHD/ASD sample (R^2^ = 0.02, OR 1.36, *P* = 1.21 × 10^−05^), and 0.4 for the ADHD sample (R^2^ = 0.045, OR 1.62, *P* = 5.75 × 10^−08^).

**Table 3:** Results of the logistic regression analyses for the ADHD PRS

ADHD PRS threshold	B	Wald p uncorrected	Bonferroni corr. Wald p	OR	Nagelkerke R^2^ PRS
ADHD/ASD sample (N = 688)					
0.01	0.243	**1.80×10**^**−05**^	**1.30×10**^**−03**^	1.275	0.013
0.05	0.274	**2.00×10**^**−06**^	**1.44×10**^**−04**^	1.316	0.016
0.1	0.278	**2.00×10**^**−06**^	**1.44×10**^**−04**^	1.321	0.017
0.2	0.287	**7.91×10**^**−07**^	**5.70×10**^**−05**^	1.333	0.018
0.3	0.304	**1.68×10**^**−07**^	**1.21×10**^**−05**^	1.355	0.020
0.4	0.297	**2.96×10**^**−07**^	**2.13×10**^**−05**^	1.346	0.019
0.5	0.297	**2.88×10**^**−07**^	**2.07×10**^**−05**^	1.346	0.019
1	0.297	**2.71×10**^**−07**^	**1.95×10**^**−05**^	1.346	0.019
ADHD sample (N = 280)					
0.01	0.337	**5.00×10**^**−06**^	**3.60×10**^**−04**^	1.401	0.024
0.05	0.356	**2.00×10**^**−06**^	**1.44×10**^**−04**^	1.428	0.026
0.1	0.401	**2.52×10**^**−07**^	**1.82×10**^**−05**^	1.493	0.031
0.2	0.454	**9.68×10**^**−09**^	**6.97×10**^**−07**^	1.574	0.039
0.3	0.472	**1.93×10**^**−09**^	**1.39×10**^**−07**^	1.603	0.043
0.4	0.482	**7.98×10**^**−10**^	**5.75×10**^**−08**^	1.620	0.045
0.5	0.479	**9.87×10**^**−10**^	**7.11×10**^**−08**^	1.614	0.044
1	0.485	**6.57×10**^**−10**^	**4.73×10**^**−08**^	1.625	0.045
ASD sample (N = 295)					
0.01	0.176	**1.45×10**^**−02**^	1	1.192	0.007
0.05	0.201	**7.33×10**^**−03**^	5.28 × 10^−01^	1.222	0.008
0.1	0.169	**2.35×10**^**−02**^	1	1.184	0.006
0.2	0.132	7.82 × 10^−02^	1	1.141	0.003
0.3	0.135	6.83 × 10^−02^	1	1.144	0.004
0.4	0.119	1.05 × 10^−01^	1	1.127	0.003
0.5	0.129	7.83 × 10^−02^	1	1.138	0.003
1	0.130	7.68 × 10^−02^	1	1.139	0.004

All models have eight PCs and sex as covariate (baseline model). Bonferroni *P*-value corrected for 72 tests. Sig. *P*-values are shown in bold. Results of the logistic regression analyses for the ASD and SCZ PRS are presented in the Supplementary Tables 3 and 4

The most lenient *P*-value threshold of *P* < 1 had a significant association in the ADHD/ASD sample, OR 1.35 (*P* = 1.9 × 10^−5^), and also in the ADHD sample OR 1.62 (*P* = 4.73 × 10^−8^). In the ASD sample none of the results remained significant after Bonferroni correction.

The ASD and SCZ PRS were not significantly associated with the ADHD, ASD, or combined ADHD/ASD status. The SCZ PRS including all SNPs (*P*-value threshold *P* < 1) showed a trend towards association in the ADHD/ASD sample (OR 1.13, *P* = 5.72 × 10^−2^) (Supplementary Tables S2 and S3 and Figs. S1 and S2).

### Sensitivity analysis: association between CBCL syndrome scales and the ADHD PRS

Based on the correlational structure in the ADHD/ASD sample (Supplemental Material Table 4) between the CBCL syndrome scale scores and the ADHD PRS *P*-value thresholds, we concluded the correlation was too low (all correlations ≤ 0.1) to warrant the linear regression analysis. Mean scores and standard deviations for the CBCL syndrome scale scores for the ADHD/ASD sample are provided in Supplementary Table 5.

## Discussion

This study investigated the associations of PRS for ADHD, ASD and SCZ, with ADHD and ASD status in a clinical child and adolescent population. As hypothesized, we found a significant association between the ADHD PRS and the combined ADHD/ASD status, and the separate ADHD status. The PRS SNP-inclusion thresholding resulted in a rise of explained variance with increasing *P*-value thresholds, showing that in addition to the GWAS significant hits, the non-significant SNPs in the ADHD GWAS also contribute to the associations with diagnostic status. Given the comorbidity between ADHD and ASD, and previously reported genetic correlations, we expected that the ADHD PRS would also be associated with ASD status, however, this association was not observed in our data. In contrast, the current results suggest a disorder specific effect of ADHD associated SNPs instead of a shared common genetic mechanism with ASD. The ADHD PRS is based on the most recent GWAS results, and explained variance up to 4.5% in our sample, which is in line with the results from the initial GWAS (Demontis and Walters 2017) who reported an explained variance of 5.5%, making it a promising PRS for further use in research on ADHD.

Contrary to our expectation, the ASD and SCZ PRS were not associated with any of the diagnostic groups. The null results for the ASD PRS are unexpected as the initial GWAS (Grove et al. 2017) reported an explained variance of 2.45% in an independent sample, and their summary statistics were used for the analysis. Given that the discovery sample size of ASD was only slightly smaller than the ADHD sample, and the SCZ sample was even larger, we do not expect that sample size alone explains these findings. Moreover, apart from sample size, power analyses usually take several parameters into account, including the heritability and population prevalence of traits, the amount of SNPs included in the GWAS, the effective number of chromosome segments, and the proportion of cases in discovery and target sample (Lee et al. 2017). In our study, the discovery and target samples were for most of the parameters similar across disorders, except for prevalence rates (ASD and SCZ have a population prevalence of 1%, and ADHD has a population prevalence of 5%).

Regarding the null result for the ASD PRS one explanation might be a difference in the diagnostic sample composition of the ASD GWAS discovery sample compared to the target ASD sample. The target sample was diagnosed with the DSM-IV and includes many cases with Asperger, and PDD-NOS diagnoses (82% of total ASD sample), which might differ from the discovery sample. Moreover, about one-third of the discovery sample were trio data (i.e. case pseudo control design), of which it has been suggested that the un-transmitted chromosomes contain increased polygenic burden, and as such the genetic signal based on these data might be decreased (Peyrot et al. 2016). Additionally, the genetic architecture of ADHD might differ from ASD, e.g., rare genetic variants might comprise a more important part of the genetic contribution to ASD (Geschwind and State 2015) compared to ADHD. With growing sample sizes, genetic discoveries will increase and become more reliable, potentially allowing the identification of rare variants.

The choice of including the SCZ PRS was based partly on the higher prevalence rate of SCZ in ASD individuals compared to the general population, a recent systematic review reports a significantly higher SCZ prevalence in ASD individuals compared to the general population (OR 3.55, 95% confidence interval (CI) 2.08–6.05, P < 0.001) (Zheng et al. 2018). If the actual SCZ prevalence rate in an ASD population resides at the lower end of the of the 95% CI the enrichment of common SCZ SNPs might not be detectable in our relatively small sample. Additionally, the genetic correlation of 0.211 between ASD and SCZ (Grove et al. 2017) and 0.122 between SCZ and ADHD (Demontis and Walters 2017) might be too small to detect the genetic overlap between the two disorders in our data. Finally, it is possible that ASD has a different genetic underpinning with more rare variants than SCZ although some overlap has been reported in rare genetic variation between ASD and SCZ (Sanders et al. 2015). Recent whole-genome sequence research on height fully recovered the heritability of this trait, meaning that next to the previously established common variants, all rare variants have been discovered (Wainschtein et al. 2019). Whole-genome sequence research into ASD, SCZ and ADHD might shed light on this issue revealing the genetic architecture of these traits.

The sensitivity analyses exploring the associations between scores on the syndrome scales of the CBCL and the ADHD PRS showed low correlations between these two measures, as such we decided not to pursue the follow-up analysis further. A reason for the low correlations can be the amount variance explained by the ADHD PRS. The explained variance of 4.5% might not be enough to give meaningful results in follow-up analysis using the CBCL in a smaller sample like “Inside out”. In addition, a diagnosis is not based solely on the CBCL results but includes careful evaluation by an experienced psychologist/psychiatrist based on a patient interview, a parent interview and if possible an evaluation by a third party like a school teacher of the child.

### Strengths and limitations

A strength of our study is the adult control sample as, in contrast to a child sample, the chance that adult individuals will receive a future ADHD or ASD diagnosis is limited compared to young individuals i.e., these disorders are usually diagnosed during childhood (Nylander et al. 2013), while DNA sequences are fixed during life.

One concern might be the difference in sex distribution between the samples, with the clinical sample consisting of 75% males and the control sample having an opposite skew in sex distribution, as this could potentially affect the observed associations between the PRS and diagnoses. However, we compared the allele frequencies between males and females in an independent sample (GoNL (Genome of the Netherlands Consortium 2014)) and found no differences. Yet, due to the skewed sex distribution we could not examine sex-PRS interactions, or sex specific associations, which would both be interesting to investigate given the higher prevalence of males in both ADHD and ASD.

We also need to take into account that the ADHD/ASD group comprises the ADHD and ASD groups and that this is no official diagnostic disorder classification. The results should be replicated in a comparable independent sample first before firm conclusions can be drawn.

Overall, despite the fact that symptoms overlap between the neurodevelopmental disorders, our study does not directly imply that the umbrella of NDD is present at the common genetic level as captured in the PRS. As the ASD and SCZ PRS do not distinguish cases from controls in any of our diagnostic samples it is possible that ADHD, ASD and SCZ have a different common genetic signature. Moreover, the results should be replicated in one or more independent samples.

A final remark can be made on the cross sectional nature of the sample. Unlike longitudinal studies, measures are available for one point in time for most of the subjects. This presents the possibility that children might receive additional diagnoses later on in life resulting in a change in diagnostic status from ADHD or ASD to the ADHD/ASD codiagnosed group, or to other comorbidities.

## Conclusions

In conclusion, the PRS of ADHD is significantly associated with the combined ADHD/ASD and ADHD status. Yet, this association is primarily driven by ADHD status, suggesting disorder specific genetic effects of the ADHD PRS. Nevertheless, it is of interest to explore the genetic predictive value of other psychiatric disorders besides neurodevelopmental disorders. Improving genetic prediction in neurodevelopmental disorders by using a multi-trait predictor instead of single-trait predictors is also an interesting option (Maier et al. 2018). Lastly, it is of interest to delve deeper into the association between the ADHD PRS and the specific emotional and behavioral problems in larger samples as those data may provide additional information on specific problems or the severity of problems within a diagnostic status.

## Electronic supplementary material

Below is the link to the electronic supplementary material.

Supplementary material 1 (DOCX 74 kb)

Supplementary material 2 (DOCX 80 kb)

Supplementary material 3 (DOCX 11 kb)

Supplementary material 4 (DOCX 15 kb)

Supplementary material 5 (DOCX 15 kb)

Supplementary material 6 (DOCX 16 kb)

Supplementary material 7 (DOCX 12 kb)
