# Supplementary material for: Psychiatric Polygenic Risk Scores as Predictor for Attention Deficit/Hyperactivity Disorder and Autism Spectrum Disorder in a Clinical Child and Adolescent Sample
Source: Behav Genet. 2019 Jul 25;50(4):203–12. doi: 10.1007/s10519-019-09965-8 (PMC7355275; doi:10.1007/s10519-019-09965-8)

Figure S1: Variance explained (Nagelkerke R^2^) by the ASD PRS under the best model, baseline with eight PCs, sex and the relevant PRS. All SNP inclusion *P-*value thresholds are shown. Given *P*-values are Bonferroni corrected, only sig. *P*-values are provided.


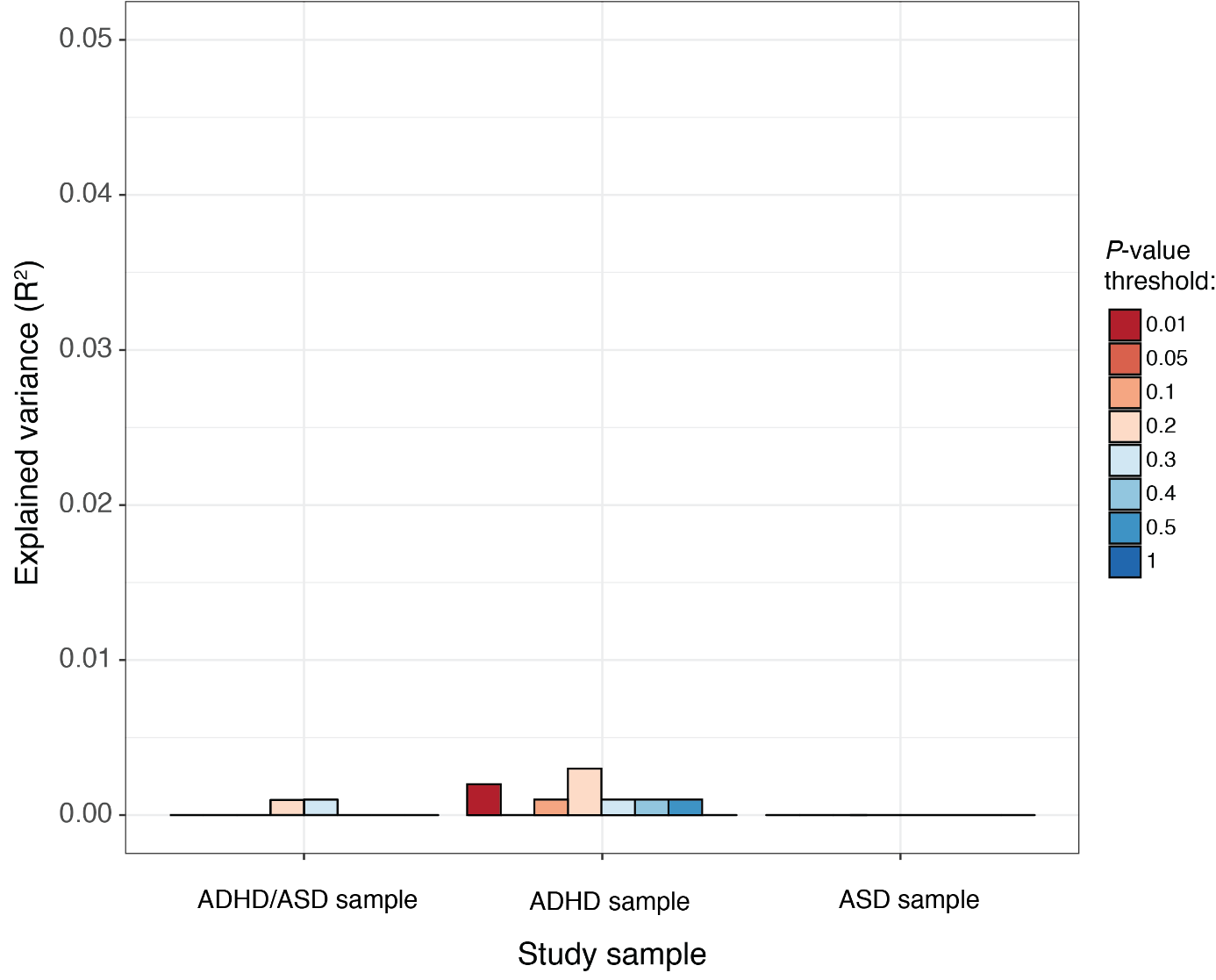

Supplement: Supplementary file 1 — Supplementary material 1 (DOCX 74 kb) [file 10519_2019_9965_MOESM1_ESM.docx]
