# Supplementary material for: Psychiatric Polygenic Risk Scores as Predictor for Attention Deficit/Hyperactivity Disorder and Autism Spectrum Disorder in a Clinical Child and Adolescent Sample
Source: Behav Genet. 2019 Jul 25;50(4):203–12. doi: 10.1007/s10519-019-09965-8 (PMC7355275; doi:10.1007/s10519-019-09965-8)
Supplement: Supplementary file 3 — Supplementary material 3 (DOCX 11 kb) [file 10519_2019_9965_MOESM3_ESM.docx]

Table S1. Amount of SNPs included per P value threshold and per disorder

| **ADHD** |  |
| --- | --- |
| **PRS P value threshold** | **amount SNPS** |
| 0.01 | 1409 |
| 0.05 | 5112 |
| 0.1 | 8930 |
| 0.2 | 15820 |
| 0.3 | 21923 |
| 0.4 | 27644 |
| 0.5 | 32980 |
| 1 | 55487 |
| **ASD** |  |
| **PRS P value threshold** | **amount SNPS** |
| 0.01 | 1233 |
| 0.05 | 4935 |
| 0.1 | 8754 |
| 0.2 | 15785 |
| 0.3 | 22277 |
| 0.4 | 28227 |
| 0.5 | 33684 |
| 1 | 57277 |
| **SCZ** |  |
| **PRS P value threshold** | **amount SNPS** |
| 0.01 | 4464 |
| 0.05 | 10214 |
| 0.1 | 14755 |
| 0.2 | 21545 |
| 0.3 | 27056 |
| 0.4 | 31997 |
| 0.5 | 36460 |
| 1 | 54524 |
