# Supplementary material for: Psychiatric Polygenic Risk Scores as Predictor for Attention Deficit/Hyperactivity Disorder and Autism Spectrum Disorder in a Clinical Child and Adolescent Sample
Source: Behav Genet. 2019 Jul 25;50(4):203–12. doi: 10.1007/s10519-019-09965-8 (PMC7355275; doi:10.1007/s10519-019-09965-8)
Supplement: Supplementary file 4 — Supplementary material 4 (DOCX 15 kb) [file 10519_2019_9965_MOESM4_ESM.docx]

Table S2.

Results logistic regression ASD PRS and case control status

| **ADHD/ASD sample** |  |  |  |  |  |
| --- | --- | --- | --- | --- | --- |
| **ASD PRS P value threshold** | **B** | **Wald p uncorrected** | **Bonferroni corr. Wald p** | **OR** | **Nagelkerke R^2^ PRS** |
| 0.01 | 0.004 | 9.38E-01 | 1 | 1.004 | 0.000 |
| 0.05 | 0.016 | 7.74E-01 | 1 | 1.016 | 0.000 |
| 0.1 | 0.045 | 4.17E-01 | 1 | 1.046 | 0.000 |
| 0.2 | 0.065 | 2.42E-01 | 1 | 1.067 | 0.001 |
| 0.3 | 0.048 | 3.98E-01 | 1 | 1.049 | 0.001 |
| 0.4 | 0.034 | 5.43E-01 | 1 | 1.035 | 0.000 |
| 0.5 | 0.033 | 5.65E-01 | 1 | 1.033 | 0.000 |
| 1 | 0.028 | 6.26E-01 | 1 | 1.028 | 0.000 |
|  |  |  |  |  |  |
| **ADHD sample** |  |  |  |  |  |
| **ASD PRS P value threshold** | **B** | **Wald p uncorrected** | **Bonferroni corr. Wald p** | **OR** | **Nagelkerke R^2^ PRS** |
| 0.01 | -0.092 | 2.09E-01 | 1 | 0.912 | 0.002 |
| 0.05 | 0.036 | 6.21E-01 | 1 | 1.037 | 0.000 |
| 0.1 | 0.078 | 2.86E-01 | 1 | 1.081 | 0.001 |
| 0.2 | 0.110 | 1.32E-01 | 1 | 1.116 | 0.003 |
| 0.3 | 0.065 | 3.78E-01 | 1 | 1.067 | 0.001 |
| 0.4 | 0.052 | 4.82E-01 | 1 | 1.053 | 0.001 |
| 0.5 | 0.062 | 4.04E-01 | 1 | 1.064 | 0.001 |
| 1 | 0.047 | 5.29E-01 | 1 | 1.048 | 0.000 |
|  |  |  |  |  |  |
| **ASD sample** |  |  |  |  |  |
| **ASD PRS P value threshold** | **B** | **Wald p uncorrected** | **Bonferroni corr. Wald p** | **OR** | **Nagelkerke R^2^ PRS** |
| 0.01 | 0.087 | 2.16E-01 | 1 | 1.09 | 0.000 |
| 0.05 | 0.000 | 9.99E-01 | 1 | 1.00 | 0.000 |
| 0.1 | 0.024 | 7.36E-01 | 1 | 1.02 | 0.000 |
| 0.2 | 0.034 | 6.36E-01 | 1 | 1.03 | 0.000 |
| 0.3 | 0.037 | 6.01E-01 | 1 | 1.04 | 0.000 |
| 0.4 | 0.012 | 8.69E-01 | 1 | 1.01 | 0.000 |
| 0.5 | 0.001 | 9.85E-01 | 1 | 1.00 | 0.000 |
| 1 | 0.010 | 8.88E-01 | 1 | 1.01 | 0.000 |

Note: Covariates included were eight PCs and sex. Multiple testing correction was applied for 72 tests
