# Supplementary material for: Psychiatric Polygenic Risk Scores as Predictor for Attention Deficit/Hyperactivity Disorder and Autism Spectrum Disorder in a Clinical Child and Adolescent Sample
Source: Behav Genet. 2019 Jul 25;50(4):203–12. doi: 10.1007/s10519-019-09965-8 (PMC7355275; doi:10.1007/s10519-019-09965-8)
Supplement: Supplementary file 5 — Supplementary material 5 (DOCX 15 kb) [file 10519_2019_9965_MOESM5_ESM.docx]

Table S3.

Results logistic regression SCZ PRS and case control status

| **ADHD/ASD sample** |  |  |  |  |  |
| --- | --- | --- | --- | --- | --- |
| **SCZ PRS P value threshold** | **B** | **Wald p uncorrected** | **Bonferroni corr. Wald p** | **OR** | **Nagelkerke R^2^ PRS** |
| 0.01 | 0.077 | 1.87E-01 | 1 | 1.080 | 0.001 |
| 0.05 | 0.090 | 1.35E-01 | 1 | 1.095 | 0.002 |
| 0.1 | 0.100 | 1.05E-01 | 1 | 1.105 | 0.002 |
| 0.2 | 0.115 | 6.95E-02 | 1 | 1.121 | 0.002 |
| 0.3 | 0.109 | 8.77E-02 | 1 | 1.115 | 0.002 |
| 0.4 | 0.112 | 8.06E-02 | 1 | 1.118 | 0.002 |
| 0.5 | 0.115 | 7.26E-02 | 1 | 1.122 | 0.002 |
| 1 | 0.122 | 5.72E-02 | 1 | 1.130 | 0.003 |
|  |  |  |  |  |  |
| **ADHD sample** |  |  |  |  |  |
| **SCZ PRS P value threshold** | **B** | **Wald p uncorrected** | **Bonferroni corr. Wald p** | **OR** | **Nagelkerke R^2^ PRS** |
| 0.01 | 0.106 | 1.71E-01 | 1 | 1.112 | 0.002 |
| 0.05 | 0.129 | 1.10E-01 | 1 | 1.138 | 0.003 |
| 0.1 | 0.125 | 1.29E-01 | 1 | 1.133 | 0.003 |
| 0.2 | 0.120 | 1.53E-01 | 1 | 1.128 | 0.002 |
| 0.3 | 0.138 | 1.05E-01 | 1 | 1.148 | 0.003 |
| 0.4 | 0.152 | 7.43E-02 | 1 | 1.164 | 0.004 |
| 0.5 | 0.153 | 7.21E-02 | 1 | 1.166 | 0.004 |
| 1 | 0.154 | 7.06E-02 | 1 | 1.167 | 0.004 |
|  |  |  |  |  |  |
| **ASD sample** |  |  |  |  |  |
| **SCZ PRS P value threshold** | **B** | **Wald p uncorrected** | **Bonferroni corr. Wald p** | **OR** | **Nagelkerke R^2^ PRS** |
| 0.01 | 0.026 | 7.25E-01 | 1 | 1.027 | 0.000 |
| 0.05 | 0.044 | 5.70E-01 | 1 | 1.045 | 0.000 |
| 0.1 | 0.085 | 2.84E-01 | 1 | 1.089 | 0.001 |
| 0.2 | 0.111 | 1.75E-01 | 1 | 1.117 | 0.002 |
| 0.3 | 0.097 | 2.41E-01 | 1 | 1.102 | 0.002 |
| 0.4 | 0.097 | 2.42E-01 | 1 | 1.102 | 0.002 |
| 0.5 | 0.101 | 2.24E-01 | 1 | 1.106 | 0.002 |
| 1 | 0.115 | 1.64E-01 | 1 | 1.122 | 0.002 |

Note: Covariates included were eight PCs and sex. Multiple testing correction was applied for 72 tests
