# Supplementary material for: Psychiatric Polygenic Risk Scores as Predictor for Attention Deficit/Hyperactivity Disorder and Autism Spectrum Disorder in a Clinical Child and Adolescent Sample
Source: Behav Genet. 2019 Jul 25;50(4):203–12. doi: 10.1007/s10519-019-09965-8 (PMC7355275; doi:10.1007/s10519-019-09965-8)
Supplement: Supplementary file 6 — Supplementary material 6 (DOCX 16 kb) [file 10519_2019_9965_MOESM6_ESM.docx]

| Table S4 |  |  |  |  |  |  |  |  |
| --- | --- | --- | --- | --- | --- | --- | --- | --- |
| correlation between ADHD PRS, several thresholds, and CBCL syndrome scales  in the ADHD/ASD sample | | | | |  |  |  |  |
|  |  |  |  |  |  |  |  |  |
| **ADHD PRS threshold** | **Anxious Depressed** | **Withdrawn Depressed** | **Somatic complaints** | **Social problems** | **Thought problems** | **Attention problems** | **Rule breaking behavior** | **aggressive behavior** |
| 0.01 | -0.08(.857) | -083(.058) | 0.018(.674) | -0.061(.167) | 0.013(.774) | 0.018(.688) | -0.004(936) | -0.034(.442) |
| 0.05 | 0.013(.762) | -0.48(.268) | 0.032(.463) | -0.061(.161) | 0.011(.809) | 0.013(.769) | -0.006(.893) | 0.001(.988) |
| 0.1 | -0.002(.964) | -0.100(.022*) | 0.017(.705) | -0.053(.226) | 0,046(.311) | 0.032(.459) | 0.043(.326) | 0.046(.291) |
| 0.2 | 0.035(.432) | -0.069(.114) | 0.045(.306) | -0.005(.917) | 0.042(.358) | 0.063(.151) | 0.066(.129) | 0.083(.059) |
| 0.3 | 0.032(.460) | -0.058(.182) | 0.054(.214) | -0.005(.907) | 0.037(.418) | 0.073(.095) | 0.065(.137) | 0.078(.075) |
| 0.4 | 0.038(.393) | -0.057(.192) | 0.045(.301) | -0.005(.907) | 0.041(.370) | 0.071(.105) | 0.071(.105) | 0.089(.044*) |
| 0.5 | 0.039(.381) | -0.054(.217) | 0.050(.254) | 0.003(.941) | 0.042(.357) | 0.067(.124) | 0.061(.165) | 0.086(.050) |
| 1 | 0.051(.248) | -0.50(.251) | 0.055(.207) | 0.014(.747) | 0.046(.319) | 0.070(.110) | 0.072(.099) | 0.090(.041*) |
|  |  |  |  |  |  |  |  |  |
| *in brackets: P-values: no multiple testing correction applied* | | |  |  |  |  |  |  |
| ** correlated significantly at the .05 level* | |  |  |  |  |  |  |  |
