# Supplementary material for: Psychiatric Polygenic Risk Scores as Predictor for Attention Deficit/Hyperactivity Disorder and Autism Spectrum Disorder in a Clinical Child and Adolescent Sample
Source: Behav Genet. 2019 Jul 25;50(4):203–12. doi: 10.1007/s10519-019-09965-8 (PMC7355275; doi:10.1007/s10519-019-09965-8)
Supplement: Supplementary file 7 — Supplementary material 7 (DOCX 12 kb) [file 10519_2019_9965_MOESM7_ESM.docx]

Table S5.

Mean scores and standard deviations of the CBCL syndrome scales for the ADHD/ASD sample. Sample sizes differ slightly per scale depending on information provided by the parents

| **CBCL syndrome scale** | **Mean** | **SD** | **N** |
| --- | --- | --- | --- |
| Anxious Depressed | 6.65 | 4.74 | 519 |
| Withdrawn Depressed | 4.71 | 3.38 | 527 |
| Somatic Complaints | 2.78 | 2.92 | 522 |
| Social Problems | 7.29 | 3.89 | 523 |
| Thought Problems | 6.34 | 4.30 | 480 |
| Attention Problems | 10.56 | 3.58 | 522 |
| Rule Breaking Behavior | 3.92 | 3.14 | 526 |
| Aggressive Behavior | 12.21 | 7.19 | 519 |
